# An evaluation of knee osteoarthritis pain in the general community—Asir region, Saudi Arabia

**DOI:** 10.1371/journal.pone.0296313

**Published:** 2024-01-11

**Authors:** Geetha Kandasamy, Dalia Almaghaslah, Mona Almanasef, Tahani Almeleebia, Rajalakshimi Vasudevan, Ayesha Siddiqua, Eman Shorog, Asma M. Alshahrani, Kousalya Prabahar, Vinoth Prabhu Veeramani, Palanisamy Amirthalingam, Saleh F. Alqifari, Vasudevan Mani, Lingala Kalyan Viswanath Reddy

**Affiliations:** 1 Department of Clinical Pharmacy, College of Pharmacy, King Khalid University, Abha, Saudi Arabia; 2 Department of Pharmacology, College of Pharmacy, King Khalid University, Abha, Saudi Arabia; 3 Department of Pharmacy Practice, Faculty of Pharmacy, University of Tabuk, Tabuk, Saudi Arabia; 4 Department of Pharmacology and Toxicology, College of Pharmacy, Qassim University, Buraydah, Saudi Arabia; 5 Department of Public Health, College of Health Sciences, Saudi Electronic University, Abha, Saudi Arabia; Sheikh Hasina National Institute of Burn & Plastic Surgery, BANGLADESH

## Abstract

**Background:**

Knee osteoarthritis (KOA) is one of the most common conditions resulting in disability, particularly in the elderly population. Osteoarthritis (OA) is the most common articular disease and the leading cause of chronic disability in the developed world.

**Objective:**

This study was carried out to evaluate knee pain in the Asir region of Saudi Arabia. An analytical cross-sectional survey design was adopted in the Asir region from April 2023 to August 2023 to assess the knee pain of the adult population using an anonymous online questionnaire.

**Results:**

Of 1234, 332 were men (26.90) and 902 were women (73.09). WOMAC index score category 55.34% (n = 683) of the subjects had a low risk (score <60), 28.68% (n = 354) had a moderate risk (score 60–80), and 15.96% (n = 197) had a high risk (score ≥81) for KOA. According to clinical criteria, 79.33% (n = 979) of the study subjects had OA. Age group, gender 2.17 (1. 67–2.82) [OR 2.17; 95% CI 1.67–2.82), family history of OA [OR 0.47; 95% CI 0.37–0.62], diabetes [OR 2.78; 95% CI 2.17–3.56], hypertension [OR 0.35; 95% CI 0.26–0.45] were significantly associated with the percentage of the WOMAC index score using the Chi-square test analysis (P<0.05). Therefore, the WOMAC index showed higher diagnostic precision with a statistically significant association [OR 9.31 CI 6.90–12.81] with a P< 0.0001.

**Conclusion:**

KOA is more common in older, obese people who have reached the age of 50 in the Asir region, and it is more prevalent in women. Alarms the need for appropriate awareness programs for better disease prevention and health outcomes for the benefit of the community through general public health programs.

## 1. Introduction

Osteoarthritis (OA) is the most common type of arthritis. It is a complex disorder that can affect the articular cartilage, bones, ligaments, and synovium. It contributes to degenerative and reparative processes and inflammation of the joint. OA may affect different body joints, both proximal and distal, most commonly occurs in the knee joint [1]. 10% of adults over the age of 60 worldwide, according to estimates from the World Health Organization (WHO), experience OA symptoms. KOA (The symptoms of KOA) symptoms are present in 10% of men and 13% of women 60 years or older. As the population ages and the obesity pandemic grows, there will probably be a rise in the number of people exhibiting symptoms of OA [2]. Symptoms and signs of OA are usually pain, stiffness, swelling that limits joint movement, crepitus, restricted movement, bone enlargement, joint effusion, and bone instability [3]. Among the various forms of arthritis, they include OA, rheumatoid arthritis, gout, fibromyalgia, and juvenile arthritis [4].

One of the most common chronic diseases that, as life expectancy increases, can cause deterioration in quality of life and increased prevalence and incidence. The illness associated with aging Articular cartilage loss and localized regions of degradation areal characteristics of OA in synovial joints. Almost 7% of people [5] have OA. A person’s quality of life may be significantly affected by pain and other OA symptoms on both a physical and psychological level. In contrast to popular belief, KOA affects the entire joint, including the articular cartilage, meniscus, ligament, and peri-articular muscle, and can be caused by a variety of pathophysiological causes. Millions of people are affected by this painful and debilitating disease.

Since OA is a very common disease, it is essential to have a precise definition of early osteoarthritis in order to adopt appropriate preventive measures that could improve the long-term quality of life of affected patients and lessen or delay the need for joint replacement interventions [6]. Degenerative joint disease (DJD) is a common term for osteoarthritis (OA). This is a misnomer, as OA involves unusual remodeling of joint structures, which is triggered by a variety of inflammatory mediators within the affected joint, rather than just a process of wear and tear. Degeneration of articular cartilage, thickening of subchondral bone, production of osteophytes, varying degrees of synovial inflammation, degeneration of ligaments and, in the case of the knee, the menisci, and hypertrophy of the joint capsule are the pathological alterations found in OA joints. Furthermore, alterations in the periarticular muscle, nerve, bursa, and local fat pad can contribute to or exacerbate OA symptoms. Varus-valgus laxity, presumably associated with ageing-related changes in the ligaments, is another factor that may contribute to the development of KOA. People with KOA frequently experience pathologic alterations of the meniscal and ligament. Injuries to the meniscus and/or joint ligaments are widely recognized to predispose to the development of OA, and magnetic resonance imaging has shown alterations even in people with no known history of joint trauma [7]. Radio frequency ablation of genicular nerves combined with a rehabilitative exercise and pharmaceutical therapies, such as acetaminophen, non-steroidal anti-inflammatory medications (NSAIDs), and opioids, are examples of non-surgical treatments for symptomatic knee OA [8, 9]. Although almost all joints are affected by OA, the knee and hip joints are the most severely affected. According to surveys, 10% to 15% of adults over 60 years of age have OA, with women more likely to have it than men. Studies have shown that the incidence of KOA is 203 per 10,000 people per year, with a global prevalence of 16% [10]. This study found that people living in Gulf cooperation council (GCC) countries have a high prevalence of OA—roughly 16.13%. In this cohort, a single study found a 3.5% OA incidence [11]. The prevalence of OA increases with age in Saudi Arabia, reaching 30.8% in those 46 to 55 years of age and 60.6% in those 66 to 75 years of age [12].

Previous research in the Kingdom of Saudi Arabia (KSA) revealed the pattern of KOA in primary care and hospital settings. However, the prevalence of KOA in the population has not been the subject of a study that covered the entire community. The aim of this study was to evaluate knee pain in the Asir region of Saudi Arabia.

## 2. Materials and methods

### 2.1 Design and setting

To evaluate knee pain during the period of five months among people in the Asir region, from 02.04.2023 to 28.08.2023, an analytical cross-sectional survey design was used. The request to participate in the study was voluntary and the survey was anonymous there were no repercussions if potential study participants declined.

### 2.2 Sample size

The sample size was calculated using the Raosoft online sample size calculator with a specified margin of error of 5% and a confidence level of error of 95% based on the approximate n = 2,211,875 total population in the Asir region of Saudi Arabia. The target sample size was selected at n = 385 to reduce the errors in the results and improve the reliability of the study. A total of 1297 students responded with a completed questionnaire. Among those 1297 questionnaires, 63 were excluded due to insufficient data.

### 2.3 Data collection

Using a chain sampling strategy, the general population of Abha, Asir region, Kingdom of Saudi Arabia was obtained. The first email was sent to students at King Khalid University and participants were included by visiting the public areas (face to face) in the different cities in the Asir region where many people of different social and economic levels were available and because of the nature of the Saudi community. The invitation to take part in the study included a request to forward the study link to relatives and any other friends who met the inclusion requirements. The data collection form’s three sections were modified from those used in earlier research. Sociodemographic information, such as sex, age, occupation, BMI, smoking, family history of OA, history of diabetes, hypertension, hypothyroidism, and hysterectomy, was intended to be collected in Section 1. The version of the WOMAC [13] OA index was used to identify patients with OA in Part 2. Furthermore, written informed consent was obtained from the respondents anonymously before participation in the study and stored confidentially.

### 2.4 Inclusion criteria

Participants in the study who were 40 years and older comprised Saudi citizens and residents. We excluded participants who did not provide written informed consent and incomplete questionnaires.

### 2.5 Western Ontario and McMaster Universities arthritis index questionnaire (WOMAC)

WOMAC is frequently used to evaluate OA of the hip and knee. It is a 24-item self-administered questionnaire that asks for pain, stiffness, and functional limitations. Walking, using stairs, lying on the bed, sitting or lying, stiffness when standing, and physical function make up the five subscales of pain. There are two subscales of stiffness: stiffness in the morning and stiffness in the afternoon. A total of 17 elements make up the physical function.

The most widely used and best validated outcome measure in individuals with KOA is the WOMAC index. The WOMAC questionnaire uses a five-point Likert scale with values ranging from 0 to 4, and each symptom is divided into four categories: none, mild, moderate, and severe. The Arabic-translated questionnaire was validated with a Cronbach alpha value of 0.93. The version of Microsoft Excel was used to add the scores for each response to each item to determine the overall score for each subscale. The Likert scale has three possible subscales: pain (0–20; five items each scored 0–4), stiffness (2 items, 0–8), and physical function (17 items, 0–8). Higher scores suggest lower physical function, stiffness, or pain. Participants might earn a maximum score of 96. The patients were divided into three risk categories according to their WOMAC scores: low risk (<60), moderate risk (60–80 score), and high risk (≥81 score). When presented as percentages, the WOMAC scores can be divided into low-risk (*≤*70%) and high risk (>70%) categories. The response is rated as unacceptable and excluded from the analysis if two or more pain elements, both stiffness elements, and four or more elements of physical function are missing. Overall WOMAC scores vary from 0 to 96, with 0 representing the highest health and 96 the worst pain condition. Poorer function is indicated by a higher WOMAC score [14]. The clinical classification criteria of the American College of Rheumatology (ACR) were also applied to all study participants as a validation of WOMAC questionnaire [15].

### 2.6 Statistical analysis

The social sciences statistical program SPSS 21.0 for Windows was used to conduct the study (IBM SPSS Statistics, New York, United States). The Chi-square test was used to determine a relationship; a significant level was established at P < 0.05. To determine the strength of the association, the odds ratio with a 95% confidence interval was determined. Analysis of the relationship between the WOMAC index and clinical criteria was performed using McNemar’s test-matched case-control study.

### 2.7 Ethical approval

The study was approved by the research committee Institutional ethical approval was obtained prior to the start of the study Approval No. ECM#2023-602- HAPO-06-B-001 dated 12.01.2023.

## 3. Results

There were a total of 1297 general public responses to our study, while 63 responses were excluded due to incomplete data. Of the 1234, 332 were men (26.90%) and 902 were women (73.09%). Among the 1234 subjects, the age group of 40 to 49 years was (n = 258; 20.90%), 50 to 59 years were (n = 214; 17.34%) 60 to 69 years were (n = 414; 33.54%) and, >70 years were (n = 348; 28.20%). employed (n = 315; 25.52%), unemployed (n = 269; 21.79%), retired (n = 144; 11.66%) housewife (n = 506; 41%). Based on BMI (13), patients are classified as normal (<18.5 kg/m2), overweight (18.5–24.9 kg/m2), obese (25–29.9 kg/m2), severely obese (30 kg / m2) was n = 173;14.01%, n = 731;59.23%, n = 234;18.96%, n = 96; 7.77% respectively. Out of 1234, 561 respondents were smokers and a family history of OA was present in n = 322; 26.09%. The history of diabetes, hypertension, hypothyroidism, and hysterectomy was n = 392, n = 307, n = 281 and n = 84, respectively. The details are given in Table 1.

**Table 1:** Sociodemographic variables of the study population.

Variables	Total (%)	Low Risk (≤70%)	High Risk (>70%)	OR (95% Cl)	P value
Gender					
Male	332 (26.90)	156	176	2.17 (1. 67–2.82)	0.0001*
Female	902 (73.09)	261	641		
Age group					
40–49 Years	258 (20.90)	181	77		0.00001*
50–59 Years	214(17.34)	71	143		
60–69 Years	414 (33.54)	83	331		
70 Years and above	348(28.20)	49	299		
BMI					
<18.5	173(14.01)	68	105		0.00001*
18.5–24.9	731 (59.23)	29	702		
25–29.9	234(18.96)	85	149		
≥30	96(7.77)	38	58		
Occupation					
Employed	315 (25.52)	105	210		
Unemployed	269 (21.79)	173	96		
Retired	144 (11.66)	99	45		
Housewife	506 (41)	353	153		0.00001*
Smoking					
Yes	561 (45.46)	300	261		
No	673 (54.53)	343	330	1.10 (0.88–1.38)	0.37
Family History of OA					
Yes	322 (26.09)	128	194		
No	912 (73.90)	528	384	0.47 (0.37–0.62)	0.0001*
Diabetes					
Yes	392 (31.76)	231	161		
No	842(68.23)	286	556	2.78(2.17–3.56)	0.0001*
Hypertension					
Yes	307 (24.87)	138	169		
No	927(75.12)	648	279	0.35 (0.26–0.45)	0.0001*
Hypothyroidism					
Yes	281(22.77)	84	197		
No	953(77.22)	619	334	0.23 (0.17–0.30)	0.0001*
Hysterectomy(n = 902)					
Yes	84	29	55		
No	818	403	415	0.54 (0.33–0.86)	0.01*

OR: odds ratio, CI: Confidence Interval *statistically significant P<0.05

BMI: Body Mass Index

To study the association between study variables and OA, WOMAC scores as a percentage [low risk (≤70%) and high risk (>70%)] were used. The independent variables are age group, BMI, occupation, history of chronic diseases such as hypertension, diabetes, trauma, family history, and hysterectomy was performed. Age group, BMI (P<0.00001), and Occupation (P<0.00001) and gender [OR 2.17; 95% CI 1.67–2.82), family history of OA [OR 0.47; 95% CI 0.37–0.62], smoking [OR 1.10; 95% CI 0.88–1.38], diabetes [OR 2.78; 95% CI 2.17–3.56], hypertension [OR 0.35; 95% CI 0.26–0.45], hysterectomy [OR 0.54; 95% CI 0.33–0.86] were significantly associated with the percentage of the WOMAC index score using Chi-square test analysis (P<0.05). Other factors were not found to be significant in Table 1.

### 3.1 Western Ontario and McMaster Universities arthritis index (WOMAC) questionnaire

A total of 3 subscales were used, that is, pain consists of 5 items; stiffness consists of 2 items; physical function consists of 17 elements. The response to each of the symptoms is classified as none, the mild, moderate, severe, and extreme experience of the symptom (five-point Likert scale response from 0 to 4**)**. The scores of each item in the WOMAC index and WOMAC scores as a percentage among the study population are shown in Table 2

**Table 2:** WOMAC Index screening tool scores among study population (n = 1234).

Variables	Number	Percentage (%)
WOMAC Index		
**Pain score (Out of 20)**		
Mean (S.D)	9.99 (5.71)	
Minimum Score	0	
Maximum Score	20	
**Stiffness Score (Out of 8)**		
Mean (S.D)	4.10 (2.43)	
Minimum Score	0	
Maximum Score	8	
**Physical Function Score (Out of 68)**		
Mean (S.D)	37.45 (9.39)	
Minimum Score	5	
Maximum Score	64	
**WOMAC Index Total Score out of 96**		
Mean (S.D)	51.59 (15.64)	
Minimum Score	21	
Maximum Score	90	
**WOMAC Index Score categories**		
Low Risk Score ≤60	683	55.34
Moderate Risk Score 61 to 80	354	28.68
High Risk (Score ≥81)	197	15.96
**WOMAC Score (as %)**		
Low Risk (≤70%)	425	34.44
High Risk (>70%)	809	65.55
**ACR Criteria**		
Yes	979	79.33
No	255	20.66

OA: Osteoarthritis, WOMAC Scores: Western Ontario and McMaster Universities Arthritis Index; ACR: American College of Rheumatology.

In addition, the percentage of the WOMAC score was compared with ACR clinical criteria of ACR. A total of 1234 subjects were positive with ACR criteria, and 447 had high-risk WOMAC scores (>70%). Thus, the WOMAC index showed higher diagnostic accuracy with statistically significant association [OR 9.31 CI 6.90–12.81] with a p-value of 0.0001 using McNemar’s test, shown in Table 3.

**Table 3:** WOMAC index compared with ACR clinical criteria for diagnosing OA among the study population using the McNemars test.

ACR criteria					
WOMAC Score	Total	Yes	No	OR (95% CI)	P Value
**High risk (>70%)**	495	447	48	9.313 (6.90–12.81)	0.0001*
**Low risk (≤70%)**	739	532	207		
**Total**	1234	979	255		

WOMAC Scores: Western Ontario and McMaster Universities Arthritis Index

OA: Osteoarthritis, OR: odds ratio, CI: confidence interval, P<0.05* Statistically significant

S.D: Standard deviation, ACR: American College of Rheumatology.

## 4. Discussion

There were a total of 1297 general public responses to our study, while 63 responses were excluded due to incomplete data. During the study period, a total of 1234 subjects were included. Of which 332 were men (26.90%) and 902 were females (73.09%). Similar results were observed by Holliday et al. and Jayaseelan et al. [16, 17]. A previous study concluded that KOA affects one fifth of adults aged 40 years and older, with a female majority in the 1.2: 1 [18]. It is interesting that women experience the effects and burden of knee osteoarthritis more than men. According to studies, osteoarthritis manifests differently in women than in males and may adversely affect specific areas of the knee. This is especially noticeable in the patellofemoral joint, where women are more likely than males to suffer from exclusive patellofemoral arthritis [18]. A previous study found that the prevalence of OA was significantly higher in women than in men [19]. Similar to our study, the increased of OA prevalence significantly increased among participants aged 60–69 years [20, 21]. Another study stated that the overall prevalence of KOA in elderly people >60 years was 28.2% [22]. In the present study based on BMI, 85.96% of respondents are above the normal body mass index. According to a study by Ryan et al., the clinical benefits of weight loss for obese people with KOA include pain relief and better quality of function [23]. In addition, the quality of life of patients with OA is significantly impacted by their pain, even when they used drugs [16]. In comparison to people without OA, the OA population also had lower social relationships, psychological well-being, and independence. To improve your health related quality of life (HRQOL), a suitable intervention is required [24].

As a result of increased BMI and prevalence of OA among older adults was increased due to decreased normal physical activities. People with a higher body mass index (BMI) are more likely to develop a metabolic syndrome, which is caused by the coexistence of several risk factors (such as hypertension and elevated lipid levels), type 2 diabetes, or coronary heart disease [25]. Additionally, earlier studies confirmed that high BMI, old age and gender, a sedentary lifestyle, inadequate or inadequate physical activity, and unhealthy eating habits are risk factors for osteoarthritis [22]. Previous study recommend that a positive relationship was found between obesity and KOA. Obesity is one of the most potential variable factors in the deterioration of OA [26].

A population-based cohort study found that both overweight and obesity increase the risk of hand, hip, and KOA, with a dose-response gradient with increasing BMI. To lower the risk of OA at these sites, healthcare practitioners should conduct prevention measures that are especially targeted at these ages [27]. Raud et al. found that the level of obesity is directly associated with the clinical consequences of KOA [28]. High physical activity was associated with low body mass index thus preventing the risk of clinical consequences to KOA. An epidemiological study conducted in the general population reported that high BMI was significantly associated with KOA and hand OA, but not with hip OA. Their study proves that obesity is a principal risk factor for the development of KOA. The clinical effects of the severity of KOA are discussed in their study for a KOA population by degree of obesity. Investigators discovered a graded relationship between the phases of obesity and the clinical effects of KOA. In fact, the findings revealed a steadily increasing relationship between clinical effects and BMI-based obesity severity. Participants with higher BMI reported more frequently experiencing feelings of depression and anxiety, had higher pain scores, and were more handicapped [29].

Another study found that obesity was a strong risk factor for KOA. In addition, they suggested reducing the weight of the patients with KOA for the treatment. The weight or obesity has been shown to increase the risk of KOA by putting a mechanical strain on weight-bearing joints. However, metabolic syndrome and obesity both increase the incidence of OA in the hand. Therefore, metabolic conditions such as diabetes or metabolic syndrome can have a general impact on joints. Type 2 diabetes mellitus (DM) may be a risk factor for OA regardless of location, according to a recent study [30]. Karine et al. stated there in their meta-analysis association between DM and OA. There may be a connection between DM, a worsening of the OA symptoms, and the severity of the OA, according to existing investigations [31]. According to the Nieves et al study, diabetes patients were more likely than non-diabetic people to have OA of the hands or knees. The possibility of KOA is greater in patients with DM compared to non-diabetic patients. Nicola Veronese et al. state that additional investigation is required to determine whether managing and preventing diabetes can affect the development and progression of OA [32]. A study by Panji et al. [33] reported that there is no difference in the severity of KOA between people with diabetes mellitus and those who have hypertension.

The prevalence of KOA was associated with hypertension and diabetes, two main risk factors for cardiovascular disease [34]. Another study supports the association between high blood pressure and KOA. This study revealed a strong relationship between hypertension and structural damage to the knee caused by OA rather than just persistent joint discomfort, particularly in women [35, 36]. A meta-analysis revealed a substantial link between high blood pressure and KOA. It is strongly supported by the causative relationship between blood pressure and OA that the vascular system plays a part in joint disease. The idea of OA as a comprehensive joint condition has become very popular in the last ten years, but the involvement of the vascular system, particularly angiogenesis, has not been thoroughly investigated [27].

Out of 1234, 561 respondents were smokers and a family history of OA was present in 26.09%. Similar to our results, another study reported that 30.7% of the population has family members with KOA [37]. According to earlier research, there is no connection between the cohort of patients undergoing arthroscopic meniscal surgery and current or past smoking [38]. In contrast to our study’s findings, several researchers found that smoking increased the risk of total knee replacement surgery in women [39]. Another study came to the same conclusion as ours: Smoking does not affect the development of OA. Nevertheless, anecdotal data suggest that cigarette smoking may be a factor in the symptoms of OA [38]. In their investigation, Kim et al. concluded that hypothyroid and hyperthyroid states were related to abnormalities on musculoskeletal ultrasound and knee arthralgia [39].

A study by Seifeldein et al. stated that pain scores, the stiffness scores, and physical function were 10.08 ±2.89 (Mean ± SD), 3.34 ± 1.72 (Mean ± SD), 26.26±9.6 (Mean ± SD) respectively. The total WOMAC score was 39.68 ± 12.83 (Mean ± SD) [40].To study the association between study variables and OA, WOMAC scores as percentages [low risk (≤70%) and high risk (>70%)] were used. The independent variables are age group, sex, family history of OA, diabetes, and hypertension were significantly associated with the percentage of the WOMAC index score using Chi-square test analysis (P<0.05). WOMAC index showed greater diagnostic accuracy with statistically significant association [OR 9.31 CI 6.90–12.81] with a p-value of 0.0001. This result was consistent with the study done by Sathiyanarayanan et al [15]. Health promotion programmes to reduce the non-communicable diseases for the general public or for communities who are more at risk of poor health outcomes. In order to give people more control over their health, health promotion typically targets behavioral risk factors like cigarette use, obesity, nutrition, and physical inactivity [41].

One of the study’s strengths is that it informs basic knowledge and highlights the frequency of knee osteoarthritis (KOA) in the Saudi community. Utilizing reliable clinical criteria to identify KOA is one of its key strengths. However, certain limitations should be addressed, such as the fact that recall bias might have existed and that information on knee injuries, family history of knee pain, etc. was self-reported. To avoid overestimating or underestimating the severity of knee pain, morbidities, and other variables, self-reported status was gathered. The cross-sectional design of the current study has a major limitation in reporting causal explanations. Additionally, due to the nature of the study design, the generalizability of the findings is limited.

### 4.1 Future recommendation

It is recommended with a larger sample size, future studies may focus on the prevalence of KOA across the entire Saudi population.

## 5. Conclusion

KOA is more common in older, obese people who have reached the age of 50 in the Asir region, and it is more prevalent in women. Alarms the need for appropriate awareness programs for better disease prevention and health outcomes for the benefit of the community through general public health programs.
